# Learning curve for laparoscopic staging of early and locally advanced cervical and endometrial cancer

**DOI:** 10.1007/s00404-013-2787-y

**Published:** 2013-03-16

**Authors:** Morva Tahmasbi Rad, Markus Wallwiener, Joachim Rom, Christof Sohn, Michael Eichbaum

**Affiliations:** 1 Department of Obstetrics and Gynecology, University of Heidelberg, Vossstraße 9, 69115 Heidelberg, Germany; 2 Department of Obstetrics and Gynecology, University of Göttingen, Göttingen, Germany

**Keywords:** Cervical cancer, Endometrial cancer, CUSUM, Learning curve, Operative time, Laparoscopic staging

## Abstract

**Background:**

Laparoscopic staging is rapidly evolving as an important surgical approach in the field of gynecology oncology. However, the specific learning curve associated with this approach remains poorly investigated. This study aimed to evaluate the learning curve for laparoscopic staging of uterine cancers.

**Methods:**

A series of 28 consecutive laparoscopic hysterectomies with or without pelvic and/or para-aortic lymph node sampling for the treatment of early and locally advanced endometrial or cervical cancer were performed between July 2008 and January 2011. The analyses of the learning curves of the institution were performed for 20 patients who had undergone pelvic lymphadenectomy and/or para-aortal lymph node sampling. The learning curve period has also been compared with the last 26 patients who received laparotomy staging (“open” group) due to the same diagnosis and by the same surgical team. To assess the short- and long-term outcomes, we used validated questionnaires to record the clinical and follow-up results, any complaints or subjective reports from the patients, and details of their quality of life. All data were collected prospectively in a database and reviewed retrospectively. The learning was evaluated using the cumulative sum (CUSUM) method.

**Results:**

The CUSUM learning curve consisted of two distinct phases: phase 1 (the initial 9 cases) and phase 2 (the subsequent cases) which presented the mastery phase, with the operative time of 397.7 ± 63.5 versus 300.6 ± 19.4 min (*p* < 0.0001). The significance of the difference between the two phases and “open” group changed in terms of number of lymph nodes retrieved, intra-operative blood loss and hospital stay. The conversion rate of phase 1 was higher than phase 2 [2 (22.2 %) respectively 1 (9 %)].

**Conclusions:**

This series confirms previous study findings concerning the feasibility and the safety of laparoscopic staging and provides information for surgeons in single centers considering adopting an endoscopic strategy to monitor the different aspects of outcomes during the implementation process for internal benchmarking. The operative outcome of laparoscopic staging intervention improves with experience. The data reported in this article suggest that after a learning curve of 9 patients, a relevant improvement at least regarding the duration of the operation can be achieved for experienced surgeons who start performing laparoscopic staging of uterine cancers. However, due to the limited number of patients as well as number of para-aortic lymph node sampling procedures, further studies are required for firm conclusions to be drawn.

## Introduction

Cervical and endometrial cancers are, respectively, second and fourth most common malignancy in women worldwide [1]. The most frequently considered treatment of patients with these two gynaecologic malignancies is total and radical hysterectomy, and depending on the histopathologic findings, the staging procedure can encompass bilateral salpingo-oophorectomy, pelvic and para-aortic lymph node dissection, omentectomy, and peritoneal biopsies which traditionally are performed with laparotomy [2]. During the last decade, laparoscopic surgery has been widely used because of its clinical advantages such as reduced post-operative pain and rapid recovery over open procedures and has also been considered as an innovation in surgical instrumentation and technology in gynecologic oncologic field of surgery [2, 3].

While an increasing number of centers are adopting minimal invasive strategies in gynecological oncology, the goal is to identify and set specific quality parameters during the process. The acquisition of competency in novel surgical techniques represents a “learning curve” [4]. Defining a learning curve for laparoscopic procedures is a complex process; however, the identification of the number of cases necessary to achieve competence is a crucial factor, which could facilitate more effective training and integrating the laparoscopic methods [4–12]. The learning curve, in addition to being a function of the surgeon’s understanding of the new technique, is improvements in support staff and peri-operative care [6, 9]. Furthermore, it is an important aspect of quality assurance in patient care [10]. A learning curve that defines the number of performed cases necessary to achieve a sufficient level is a graphic representation of the relationship between the experiences of performing procedure with the outcome variables that are of clinical interest [7].

Only a limited number of publications report on multi-dimensional assessment of the learning curve, including operating time, conversion rate, intra-operative complications, and post-operative complications in a new integrated surgical technique [13–17].

To the best of our knowledge, no study in the field of laparoscopic staging in cervical and endometrial cancers has used cumulative sum analysis (CUSUM), which transforms raw data into running total data deviations from their group mean, enabling investigators to visualize the data for trends not discernible with other approaches [11, 12]. In our institute, at the beginning of integrating laparoscopic staging, high clinical outcomes including tumor free resection boarders, complication-less operations and minimal bleeding have been set as primary aims in all the patients. Due to this fact, we have only investigated operating time as the main evaluating factor in our learning curve and investigated the changes in goal outcomes during the learning curve. This study aimed to analyze the initial learning curve for laparoscopic staging in cervical and endometrial carcinoma using CUSUM methodology.

## Materials and methods

In July 2008, it has been started to use laparoscopic intervention in patients with early stages of endometrial or cervical cancers at the department for Gynecology and Obstetrics of the University of Heidelberg Medical School. Between July 2008 and January 2011, 28 consecutive patients underwent laparoscopic hysterectomy with or without pelvic (PEL) and/or para-aortic lymph node sampling (PAS) for the treatment of early and locally advanced endometrial or cervical cancer at our department. In this retrospective study, the patients who underwent laparoscopic staging in the mentioned period of time (“laparoscopy” group) have been compared with the last consecutive 28 patients who received laparotomy staging (“open” group). All these patients were preoperatively diagnosed as early stages of endometrial or cervical cancer and have been operated by the same team of surgeons. Demographic data, intra-operative findings, procedures details, post-operative parameters, morbidities, and outcomes were collected prospectively in our database and reviewed prospectively. To assess the short- and long-term outcomes, we used validated questionnaires to record the clinical and follow-up results, any complaints or subjective reports from the patients, and details of their quality of life. All patients have been followed up for 12–28 months.

The operative time was measured from skin incision to wound closure at completion. Conversion to open surgery was defined as any case that could not be completed laparoscopically as planned and that involved extension of the incision more than necessary for specimen extraction or creation of an alternative incision to complete the procedure. A complication was defined as any deviation from the normal intra- or post-operative course. The post-operative complication has been considered as any morbidity and readmission within 30 days after surgery. To classify the severity of the complications, we used the Dindo–Clavien classification consisting of five grades and two sub-grades.

Surgical technique in the laparoscopic group was completed as follows: under general anesthesia, the patients were placed in the dorso-lithotomy position. Mostly, the 4-puncture technique was used, a direct primary trocar was placed through the umbilicus, and the peritoneal cavity was inspected. The round ligaments were transected bilaterally, and paravesical and pararectal spaces were created by blunt dissection. In cases where para-aortic lymph node sampling was performed, one more 12-mm trocar was inserted in the left upper abdominal quadrant, and camera and surgeon’s positions were changed. Pelvic lymph node dissection was extensively performed up to the parametrial area, and para-aortic lymph node sampling was performed up to the level of the renal artery with the sampling of para-caval, para-aortic lymphatic tissue as well as lymphatic tissues between vena cava and aorta.

A radical hysterectomy was performed as described in the following: the uterine artery was divided at its origin from the hypogastric artery. The ureter was then unroofed and dissected from the parametria and the bladder was dissected further inferiorly. Then, the anterior and posterior leaves of the vesico-uterine ligaments were dissected. The uterosacral and cardinal ligaments were isolated and resected as close as possible to the pelvic side walls, depending on whether a type II or type III radical hysterectomy was performed. The vaginal wall was incised circumferentially with a needle coagulator. The specimen was removed. In cases where a type I radical hysterectomy or a simple total laparoscopic hysterectomy was undertaken, there was no parametrial resection.

Demographic data, operative outcomes, and complications were analyzed by the Pearson Chi square test, Fisher’s exact test and Mann–Whitney *U* test. A two sided *p* value of level of significance was defined at *p* value <0.05. The statistical analyses were performed using Statistical Package for Social Sciences (SPSS version 15.0 for Windows; Chicago, IL, USA).

Regarding the importance of the sufficiently performed lymphadenectomy and its considerable effect on the complexity of gynecology-oncological operations, the patients without lymphadenectomy have been compared separately; the learning curve has only been investigated in patients received PEL and/or PAS.

We used the cumulative sum (CUSUM) technique for quantitative assessment of the learning curve in laparoscopic operations which included PEL or PAS. The CUSUM is the running total of differences between the individual data points and the mean of all the data points. The CUSUM was used to assess the operative time, to calculate the CUSUM, the cases were arranged chronologically. CUSUM (SN) was defined as ${\text{SN}} = \sum \left( {X_{i} - X_{0} } \right)$, when *X*_*i*_ was an individual attempt and *X*_0_ was the mean operative time for all the cases. After each attempt, scores were sequentially added to the cumulative scores and was then plotted graphically. This study was approved by the Ethics committee of the Medical faculty of the University of Heidelberg.

## Results

During the study period of 29 months, 28 patients underwent laparoscopic staging of early stages of either cervical or endometrial cancer. All patients in both groups have been followed up between 12 and 28 months.

The patients in “laparoscopy” group have received the following intervention: 8 (28.6 %) hysterectomy with or without adnexectomy/ovariopexy, 13 (46.4 %) hysterectomy with PEL with or without adnexectomy/ovaropexy and 7 (25 %) hysterectomy with PEL and PAS with or without adnexectomy/ovaropexy. These staging operations were done in 17 patients with cervical cancer and 11 patients with endometrial cancer. The used technique in the patients with cervical cancer was laparoscopic radical hysterectomy. From 11 patients with endometrial cancer, 5 had undergone total laparoscopic hysterectomy and 6 had undergone laparoscopic assisted vaginal hysterectomies.

The last 28 patients who received laparotomy staging (“open” group) of early stages of cervical and endometrial cancer were as follows: 2 (7.1 %) hysterectomy ± adnexectomy/ovaropexy, 19 (67.9 %) hysterectomy with PEL ± adnexectomy/ovaropexy and 7 (25 %) hysterectomy with PEL and PAS ± adnexectomy/ovaropexy.

Generally, our “laparoscopy” group was significantly younger than our “open” group (48.8 ± 11.2 vs. 55.6 ± 10.10; *p* = 0.02) without any considerable differences in BMI (26.5 ± 6.0 vs. 26.73 ± 4.9). Previous operations were reported in 5 (17.9 %) patients in “laparoscopy” group (2 appendectomies, 1 cholecystectomy, 1 cesarean section (two times), 1 liver transplantation (two times)) and 5 (17.9 %) in “open” group (2 appendectomies, 2 cholecystectomies, 1 cesarean section). One patient received laparoscopic staging after two liver transplantations, which has been reported previously [18]. The operation time in the “laparoscopy” group (296.7 ± 94.4 min) was significantly longer than “open” group (221.9 ± 64.8 min) (*p* = 0.001). The “open” group had a higher blood loss in comparison to our “laparoscopy” group (231.2 ± 81.6 vs. 131.3 ± 103.8 cc; *p* < 0.0001) which was compatible with delta hemoglobin levels (2.7 ± 0.8 vs. 1.9 ± 0.6 cc; *p* = 0.002).

### Learning curve

**Fig. 1:**
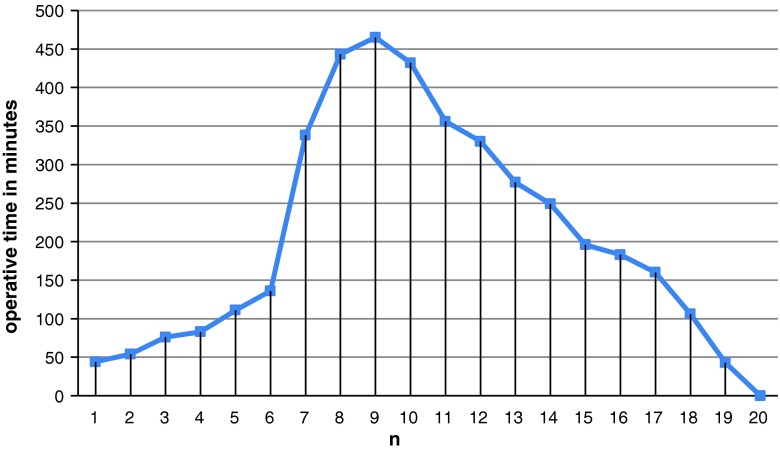
Cumulative sum for operative time (CUSUM) plotted against case number

The CUSUM learning curve is shown in Fig. 1. This curve was observed to consist of two different phases: phase 1 (the initial 9 cases) and phase 2 (the last 11 case) (Table 1).

**Table 1:** Operating time according to number of the cases in 2 phases of learning curve

Phase 1 patients’ number	Operating time (min)	Phase 2 patients’ number	Operating time (min)
1	387	10	310
2	353	11	267
3	365	12	317
4	350	13	290
5	371	14	315
6	368	15	290
7	545	16	330
8	448	17	320
9	365	18	289
		19	280
		20	300

A comparison of various parameters between these two phases identified by CUSUM analysis is summarized in Table 2. Each of these two phases has also been compared with the “open” group.

**Table 2:** Demographic and peri-operative data, comparison between two laparoscopic phases and “open” group

	“Laparoscopy” group		“Open” group (26 patients)	*p* value		
	Phase 1 (9 patients)	Phase 2 (11 patients)		Phase 1 versus phase 2	Phase 1 versus “open” group	Phase 2 versus “open” group
Age (mean ± SD, years)	41.22 ± 6.7	54.7 ± 11.7	54.7 ± 9.9	0.007	0.001	0.9
BMI (mean ± SD, kg/m^2^)	22.5 ± 2.5	27.1 ± 5.5	26.1 ± 7.6	0.03	0.01	0.85
Previous operations	1 (appendectomy)	2 (1 appendectomy, 1 cholecystectomy)	3 (1 appendectomy, 2 cholecystectomy)	–	–	–
Conversion	2 (22.2 %) intra-operative complications	1 (9 %) unexpected findings	–	–	–	–
Intra-operation complications	2 (22.2 %) grade IIIb	1 (9 %) grade I	1 (3.8 %) grade IIIb			
Number of harvested PEL nodes (mean ± SD)	17.3 ± 7.7	22.5 ± 7.2	30.2 ± 15.8	0.14	0.02	0.13
Number of harvested PAS nodes (mean ± SD)	–	2 ± 1.3 (7 patients)	5.2 ± 3.1 (7 patients)	–	–	0.04
Number of total harvested lymph nodes (mean ± SD)	17.3 ± 7.7	24.4 ± 5.5	31 ± 16.6	0.04	0.03	0.2
Blood loss (cc)	222.2 ± 148.7	79.55 ± 15.6	249 ± 79.2	0.005	0.52	0.001
Delta Hb	2.3 ± 0.4	1.7 ± 0.6	2.7 ± 0.8	0.02	0.17	0.001
Post-operation complications^a^	2 grade I 1 grade IIIb (1 vaginal cuff hematoma)	2grade I 1 grade IIIb (wound colloid)	7 grade I 3 grade IIIb (1 wound hematoma, 2 wound dehiscence)	–	–	–
Operation time (min)	397.7 ± 63.5	300.6 ± 19.4	228.7 ± 61.5	<0.0001	<0.0001	0.001
Hospital stay (days)	14.8 ± 6.9	9.6 ± 4.0	13.4 ± 4.2	0.05	0.5	0.01

*PEL* pelvic lymphadenectomy, *PAS* para-aortal lymph node sampling

^a^According to Dindo-Clavien classification

In phase 1 comparing to phase 2 as well as “open” group, younger patients with lower BMI have been operated. In phase 1, we have had 2 (22.2 %) grade IIIb intra-operative complications (1 bladder injury, 1 ureteral injury) which ended to conversion to laparotomy. In phase 2, there were only 1 (9 %) grade I intra-operative complication (partial obturator nerve injury) and 1 (9 %) conversion to laparotomy which was due to impossibility of performing lymphadenectomy because of considerable intraabdominal obesity. Total number of harvested lymph nodes has significantly increased from phase 1 to phase 2 (17.6 ± 8.4 vs. 24.4 ± 5.5, *p* value = 0.04), but still lower than “open” group (31 ± 16.6, *p* < 0.05). The blood loss in phase 1 was similar to “open” group (222.2 ± 148.7 vs. 249 ± 79.2 ml, *p* < 0.05) but decreased significantly in phase 2 (79.6 ± 15.6 ml, *p* = 0.005). The operation time reduced significantly from phase 1 to phase 2 (397.7 ± 63.5 vs. 300.6 ± 19.4 min, *p* value <0.0001) but was still higher than “open” group (228.7 ± 1.5 min, *p* = 0.001). The negative resection margin was achieved in all the patients in 3 studied groups. The details of histopathologic findings have been summarized in Table 3.

**Table 3:** Histopathology details of studied groups

	“Laparoscopy” group		“Open” group (26 patients)
	Phase 1 (9 patients)	Phase 2 (11 patients)	
Cervical carcinoma	8 (88.8 %)	5 (45.4 %)	12 (46.2 %)
Endometrial cancer	1 (11.1 %)	6 (54.5 %)	14 (53.8 %)
Histology			
Adenocarcinoma	1 (11.1 %)	6 (54.6 %)	16 (61.5 %)
Squamous	8 (88.8 %)	5 (45.5 %)	10 (38.5 %)
Differentiation or grade			
Well (G1)	0	1 (9 %)	4
Moderate (G2)	5 (55.5 %)	4 (36.4 %)	15
Poor (G3)	4 (44.4 %)	6 (54.5 %)	7
Nodal involvement			
Yes	0	1 (one pelvic node)	1 (one pelvic node)
No	9 (100 %)	10 (90.9 %)	25 (96.2 %)
Lymphovascular permeation			
Present	1 (11.1 %)	2 (18.2 %)	2 (7.7 %)
Not present	8 (88.8 %)	9 (81.8 %)	23 (92.3 %)

FIGO stage^a^	Total	CC	EC	Total	CC	EC	Total	CC	EC
IA	3 (33.3 %)	2	0	2 (18.2 %)	0	2	10 (38.5 %)	4	6
IB	5 (55.6 %)	4	1	8 (36.6 %)	5	3	14 (52.2 %)	8	6
II	1 (11.1 %)	1	0	0	0	0	2 (7.7 %)	1	1
IIIA	0	0	0	1 (9.1 %)	0	1	0	0	0

*CC* cervical cancer, *EC* endometrial cancer

^a^The resection margin in all the patients was negative (R0)

Post-operation complications reduced from phase 1 (44 %) to phase 2 (36 %) with no significant differences between two phases as well as open group (38 %). The grade I reported complication consisted of the patients’ complaints about sensation, movement and edema in legs which did not need any interventions. Although hospital stay showed no significant difference between phase 1 and “open” group (14.8 ± 6.9 vs. 13.4 ± 4.2 days), reached a considerable better status in phase 2 (vs. 9.6 ± 4.0 days, *p* value = 0.01).

In terms of quality of life and post-operative outcomes, in phase 1 only the patients’ cosmetic satisfaction was significantly better than “open” group (*p* = 0.007), this situation changed to significantly better outcomes in all parameters including: duration to have pain after surgery (*p* = 0.01), post-operative pain strength (*p* = 0.001), time to return to normal life (*p* = 0.003) as well as cosmetic satisfaction (*p* = 0.03) (Table 4). No mortality due to the gynecologic oncology has been reported in these series.

**Table 4:** Quality of life and post-operative outcome, comparison between laparoscopic phases and “open” group

	“Laparoscopy” group		“Open” group *n* (%)	*p* value		
	Phase 1 *n* (%)	Phase 2 *n* (%)		Phase 1 versus phase 2	Phase 1 versus “open” group	Phase 2 versus “open” group
Post-operation pain				0.2	0.4	0.01
Until 1 week	4 (44)	8 (72.7)	6 (23.1)			
Until 1 month	3 (33)	3 (27.3)	14 (53.8)			
Until 1 year	2 (22)	0	6 (23.1)			
Pain strength				0.1	0.3	0.001
0–3	5 (55.5)	10 (90.9)	7 (26.9)			
4–6	3 (33)	0	12 (46.2)			
7–9	1 (11)	1 (9.1)	7 (26.9)			
Return to normal life in				0.01	0.3	0.003
2 weeks	0	8 (72.7)	3 (11.5)			
4 weeks	4 (44)	1 (9.1)	5 (19.2)			
6 weeks	2 (22)	1 (9.1)	11 (42.3)			
>6 weeks	3 (33)	1 (9.1)	7 (26.9)			
Cosmetic satisfaction				0.6	0.007	0.03
High	6 (66.7)	6 (54.5)	4 (15.4)			
Fair	3 (33)	4 (36.4)	12 (46.2)			
Low	0	1 (9.1)	10 (38.5)			

## Discussion

The adaptation of new technology, due to moral and ethical concerns involving patient safety, is a challenge particularly in the practice of medicine [4, 5]. The learning curve is a graphic representation of the temporal relationship between the surgeon’s mastery of a specifically assigned task and the chronological number of cases performed [6–8]. Although learning theorists often disagree about what learning curve is, they agree that its affects are clearly cumulative and may therefore plotted as a curve [10–12]. By cumulative it is meant that somehow the effect of experience carry over to aid later performance. This property is fundamental to the construction of learning curves [4–10]. The CUSUM technique is a method adopted by the medical profession in the 1970s to analyze the learning curve for surgical procedure [11, 12] and transforms raw data into running total data deviation from their group mean, enabling investigators to visualize the data for trends not discernible with other approaches [9–12]. Defining a learning curve for laparoscopic procedures is a complex process; however, the identification of the number of cases necessary to achieve competence is a crucial factor, which could facilitate more effective training and integrating the laparoscopic methods.

We chose CUSUM analysis because meaningful conclusion cannot be drawn from raw data plotted by chronological cases [11, 12]. We used this method to investigate the learning curve in staging in early stages of cervix and endometrial carcinomas. However, to date few series have reported the learning curve associated with laparoscopic surgery in gynecology [19–21] and even fewer in gynecology oncology [22, 23]. Moreover, to the best of our knowledge, none of them have used CUSUM analysis. Our study using CUSUM analysis identified 2 unique phases of the learning curve in the field of laparoscopic staging: phase 1 found to require 9 cases which can represent the initial learning curve phase and phase 2 represent the mastery phase, with a reduction in operating time.

Publications investigating the learning curve in laparoscopic interventions in gynecology have performed their analysis used on chronological cases that split into predefined segments with univariate analysis performed to compare means across segments [19–23]. For instance, Wattiezet et al. [19], investigating the learning curve of total laparoscopic hysterectomy in benign uterine diseases, have compared two groups chronologically based on using a new uterine manipulator. In a similar study on the learning curve of total laparoscopic hysterectomy, Garrett et al. [20] divided the patients into 3 groups with 40 patients. Paek et al. [21] have published the learning curve for single-port access total laparoscopic hysterectomy, by dividing the first 100 consecutive cases into 5 groups with 20 patients. Holub et al. [22] have reported the learning curve of laparoscopic surgery in women with endometrial cancer by arranging the patients chronologically and dividing them into 3 groups. Chong et al. [23] have published the learning curve of laparoscopic radical hysterectomy with pelvic and/or para-aortic lymphadenectomy in early and locally advanced cervical cancer, with splitting the patients into two groups with 50 patients.

Normally, a higher complications rate is expected during a surgeon’s learning curve [7]. It is demonstrated that gradually, the significant decrease of conversion to laparotomy and major intra-operative complications could be seen [19, 22, 23] among these complications—excessive hemorrhage and urinary tract injury are the prominent ones [19]. In our study, the conversion rate decreased from 22.2 % in phase 1–9 % in phase 2; while reasons of conversions in phase 1 was intra-operative complications, in phase 2 it was due to the unexpected finding (intra-operative difficulties including ventilation problems due to high adiposity) restricting the operating technique. The intra-operative complication rate and their complexity decreased from phase 1 to phase 2, in this regard phase 2 has also had better situation comparing to “open” group. Interestingly, the urinary tract injuries only happened in phase 1. Blood loss and the transfusion rate are also the variables that have been emphasized to decrease in the learning curve [19, 21–23].

In addition, longer operating time is expected during the learning curve as well [6]. Although the above mentioned studies [19–23], reporting learning curve in gynecology laparoscopy, have investigated different fields, all of them have reported a significant decline in operating time [19, 22, 23]. In our series, we have demonstrated a decline of approximately 90 min. Chang et al. [23], who have reported learning curve in laparoscopic staging of cervical cancer, have also shown a decrease of 100 (325 vs. 225, *p* < 0.001) minutes in operating time. They have also demonstrated the significant increase of the acquired number of PEL (15.8 vs. 26.9, *p* < 0.001). Although we have also shown an increase in harvested PEL nodes (17.3 vs. 22.5), this difference was not significant which is similar to the study done by Holub et al. (12.4 vs. 15.4) [22].

In our study, like other reports [22, 23] the number of patients who underwent para-aortic lymph node sampling was too small and their distribution was asymmetrical for comparison. This can explained by the fact that generally para-aortic lymphadenectomy is a more challenging procedure than pelvic lymphadenectomy and thus, achieving acceptable outcomes in patients needing para-aortic lymphadenectomy probably requires a longer learning curve with special training programs, which must be investigated in future studies.

Other important aspect of introducing a minimal invasive surgery is the decrease of hospital stay. All the studies reporting the learning curve have shown a significant decrease in hospital stay [19–23]. Although in our series, the hospital stay in phase 1 was similar to “open” group, in phase 2 we have seen a significant shorter hospital stay in comparison to “open” group. Between two phases, the decrease of 5 days hospital stay can be seen and in the other study, this has been reported about 3 days [23].

Garret et al. [20] have reported no significant changes during the learning curve, in the terms of complications, operating time and bleeding, which could be due to very high number of teaching cases in this series.

Holub et al. [22] demonstrated that in cases without lymphadenectomy, there was no significant difference in operating time, estimated blood loss, rate of conversion to laparotomy, operative complications and length of hospital stay among the compared groups which is similar to our findings.

In terms of quality of life and post-operative outcomes, we have demonstrated that in cosmetic satisfaction, even in the phase 1 in comparison to “open” group, we can expect significantly better outcomes. In phase 2, all the aspects including post-operative pain, pain strength and time to return to normal life as well as cosmetic satisfaction have significant better outcomes (Table 4).

This study demonstrated the feasibility and safety of performing laparoscopic staging. We have shown that an initial period is usually necessary for surgeons to become proficient in this procedure. Our data suggest that after a learning curve phase of 9 cases, the surgeon may achieve a higher level of competence and consider offering this approach to patients presenting with more complicated cases. Finally, it could be argued that the good outcome reported in this article is partly reflecting the previous laparoscopic experience. Undeniably, these facts may have an impact on the learning curve as well.

To our knowledge, this is the first series to evaluate the learning curve of laparoscopic staging using a CUSUM method.

However, due to the limited number of patients as well as number of para-aortic lymph node sampling procedures, further studies are required for firm conclusions to be drawn.

## References

[1] Siegel R, Naishadham D, Jemal A (2012) Cancer statistics, 2012. CA Cancer J Clin 62(1):10–29. doi: 10.3322/caac.20138 [Epub 2012 Jan 4] 10.3322/caac.20138 22237781

[2] Martin-Hirsch PL, Wood NJ (2011) Cervical cancer. Clin Evid (Online) 2011 Jul 27 PMC3217784 21791123

[3] Gray HJ (2008). Primary management of early stage cervical cancer (IA1-IB) and appropriate selection of adjuvant therapy. J Natl Compr Canc Netw.

[4] Subramonian K, Muir G (2004). The ‘learning curve’ in surgery: what is it, how do we measure it and can we influence it?. BJU Int.

[5] Guillonneau BD (2005). The learning curve as a measure of experience. Nat Clin Pract Urol.

[6] Aggarwal R, Moorthy K, Darzi A (2004). Laparoscopic skills training and assessment. Br J Surg.

[7] Morgenstern L (2005). Warning! Dangerous curve ahead: the learning curve. Surg Innov.

[8] Buchmann P, Dinçler S (2005). Learning curve–calculation and value in laparoscopic surgery. Ther Umsch.

[9] Verdaasdonk EG, Stassen LP, van der Elst M, Karsten TM, Dankelman J (2007). Problems with technical equipment during laparoscopic surgery. An observational study. Surg Endosc.

[10] Ramsay CR, Grant AM, Wallace SA, Garthwaite PH, Monk AF, Russell IT (2000). Assessment of the learning curve in health technologies. A systematic review. Int J Technol Assess Health Care.

[11] Chaput de Saintonge DM, Vere DW (1974). Why don’t doctors use cusums?. Lancet.

[12] Wohl H (1977). The cusum plot: its utility in the analysis of clinical data. N Engl J Med.

[13] Buchs NC, Pugin F, Bucher P, Hagen ME, Chassot G, Koutny-Fong P, Morel P (2011) Learning curve for robot-assisted Roux-en-Y gastric bypass. Surg Endosc 2011 Nov 2. [Epub ahead of print] 10.1007/s00464-011-2008-3 22044973

[14] Schauer P, Ikramuddin S, Hamad G, Gourash W (2003). The learning curve for laparoscopic Roux-en-Y gastric bypass is 100 cases. Surg Endosc.

[15] Okrainec A, Ferri LE, Feldman LS, Fried GM (2011). Defining the learning curve in laparoscopic paraesophageal hernia repair: a CUSUM analysis. Surg Endosc.

[16] Bokhari MB, Patel CB, Ramos-Valadez DI, Ragupathi M, Haas EM (2011). Learning curve for robotic-assisted laparoscopic colorectal surgery. Surg Endosc.

[17] Mohr CJ, Nadzam GS, Curet MJ (2005). Totally robotic Roux-en-Y gastric bypass. Arch Surg.

[18] Tahmasbi Rad M, Wallwiener M, Schemmer P, Schott S, Sohn C, Rom J, Eichbaum M (2012). Laparoscopically assisted vaginal hysterectomy in a patient with micro-invasive cervical cancer after two liver transplantations. J Obstet Gynaecol Can.

[19] Wattiez A, Soriano D, Cohen SB, Nervo P, Canis M, Botchorishvili R, Mage G, Pouly JL, Mille P, Bruhat MA (2002). The learning curve of total laparoscopic hysterectomy: comparative analysis of 1647 cases. J Am Assoc Gynecol Laparosc.

[20] Garrett AJ, Nascimento MC, Nicklin JL, Perrin LC, Obermair A (2007). Totallaparoscopic hysterectomy: the Brisbane learning curve. Aust N Z J Obstet Gynaecol.

[21] Paek J, Kim SW, Lee SH, Lee M, Yim GW, Nam EJ, Kim YT (2011). Learning curve and surgical outcome for single-port access total laparoscopic hysterectomy in 100 consecutive cases. Gynecol Obstet Invest.

[22] Holub Z, Jabor A, Bartos P, Hendl J, Urbánek S (2003). Laparoscopic surgery in women with endometrial cancer: the learning curve. Eur J Obstet Gynecol Reprod Biol.

[23] Chong GO, Park NY, Hong DG, Cho YL, Park IS, Lee YS (2009). Learning curve of laparoscopic radical hysterectomy with pelvic and/or para-aortic lymphadenectomy in the early and locally advanced cervical cancer: comparison of the first 50 and second 50 cases. Int J Gynecol Cancer.

